# Diet-induced changes in bacterial communities in the jejunum and their associations with bile acids in Angus beef cattle

**DOI:** 10.1186/s42523-020-00051-7

**Published:** 2020-09-16

**Authors:** Jianan Liu, Fang Liu, Wentao Cai, Cunling Jia, Ying Bai, Yanghua He, Weiyun Zhu, Robert W. Li, Jiuzhou Song

**Affiliations:** 1 Department of Animal & Avian Sciences, University of Maryland, College Park, MD 20742 USA; 2 United States Department of Agriculture, Agriculture Research Service, Animal Genomics and Improvement Laboratory, Beltsville, MD 20705 USA; 3 College of Food Science and Engineering, Ocean University of China, Qingdao, China; 4 Department of Human Nutrition, Food and Animal Sciences, University of Hawai’i at Manoa, Honolulu, HI 96822 USA; 5 College of Animal Science, Nanjing Agricultural University, Nanjing, China

**Keywords:** 16S rRNA gene sequencing, Angus beef cattle, Grass-fed beef, Jejunal bacteria, Bacterial signatures, Bile acids

## Abstract

**Background:**

The small intestine, while serving as the main absorption organ, also possesses a unique bacterial environment and holds the critical function of conversion of primary bile acids. Bile acids are, in turn, able to regulate bacterial composition and promote the growth of bacteria that convert primary bile acids to secondary bile acids. However, in beef cattle, few studies have explored the influence of diets on jejunal bacterial communities and examined its relationships with bile acids. Here, we examined the impact of grain- and grass-based diets on jejunal and fecal bacterial communities’ composition and investigated possible association of bacterial features with bile acids.

**Results:**

We demonstrated that the influences of diets on intestinal bacteria can be observed in young beef cattle after weaning. A significantly higher level of microbial diversity was documented in feces of grass-fed cattle comparing to grain-fed cattle. Top 20 important genera identified with random forest analysis on fecal bacterial community can be good candidates for microbial biomarkers. Moreover, the jejunal bacteria of adult Angus beef cattle exhibited significant differences in microbial composition and metabolic potential under different diets. Global balances and bacteria signatures predictive of bile acids were identified, indicative of the potential association of bacterial features with bile acids.

**Conclusions:**

The findings from this study provided novel insights into the relationships between jejunal bacteria and bile acids under different diets in Angus beef cattle. Our results should help us gain a better understanding of potential health benefits of grass-fed beef.

## Background

Beef is a flavorful meat product that contains high-quality protein and various nutrients, which makes it popular in the meat market. In the beef cattle feeding industry, while grain-fed method feeds young cattle in feedlots with high-grain diets, grass-fed mode allows cattle to graze naturally with grass-based diets in the pasture. As the demand for healthy and palatable beef is increasing worldwide, grass-fed beef products gradually attract more attention in the meat market; a growing group of consumers is willing to pay a higher unit price for grass-fed beef than grain-fed beef [1]. Besides animal behavior aspects, diet component is one of the main differences between grain-fed and grass-fed methods to determine cattle performance [2]. One critical influence of diets is that diets primarily influence gut bacterial structures and drive microbial adaption in animals and humans [3]. The composition and function of gut bacteria in ruminant species are critical. Bacteria are the dominant kinds of microbes in the ruminant gut, which provide a significant contribution of metabolomic activities on cattle [4]. So far, many gut microbiome studies have focused on rumen bacteria to assess gut environment and meat performance of cattle, but roles of microbes in small intestine and colon are less explored. However, different segments of ruminant gastrointestinal tract possess unique ecological and microbial characteristics [5, 6]. After the digestive activity in the upper GI tract, peristalsis and a portion of undigested fibrous residuals together push digesta downward, to enter the small intestine and be in contact with villi of the intestinal epithelium where absorption happens. The small intestine is the main gut segment for the absorption of glucose, amino acids, fatty acids, and glycerol with the aid of bile and enzymes [4]. Because of the limited contact with the outside environment, low oxygen level, and little development time here, the small intestine of cattle GI tract is the segment where abundance and diversity of microorganisms are significantly reduced compared to rumen and hindgut (cecum and colon) in ruminant species [5, 7–9]. Although the abundance is relatively low, the bacteria in the small intestine played essential roles to aid digestion, including the conversion of primary bile acids, and the protection against pathogens in both human and animals [10, 11]. Importantly, by microbial actions in the small intestine, primary bile acids are mediated to produce secondary bile acids, deoxycholic acid (DCA) and lithocholic acid (LCA), which are conjugated and finally reabsorbed by the enterohepatic circulation for the recycling in liver to regulate digestion [12, 13]. Diet, as the primary determinant of the gut microbiome, may also post significant influences on bacterial structure of small intestine in cattle. Therefore, the small intestine bacteria and their relationships with bile acids are worth the exploration.

Previous studies of cattle microbial communities throughout the GI tract demonstrated compositional differences of the bacteria in the small intestine compared to other segments in both dairy and beef cattle [5, 14]. But not much information could be found to illustrate the influence of diet on the jejunal bacteria in beef cattle research. In this study, we hypothesized that bacterial compositions in the intestine are associated with diet components. We used Wye Angus beef cattle from the University of Maryland (UMD) Wye Angus farm. This herd has been closed to any animal importation for almost 70 years to produce homogeneous progeny [15]. The calves were treated and raised in the same way until weaning. After weaning, the calves were randomly assigned to either grass or grain-fed style to reach market weight and get slaughtered. Our previous studies using the same animals already pointed out several advantages of the grass-fed method over the grain-fed method [16, 17]. In this study, we would like to probe further from the aspect of the gut bacteria to explore the potential roles of bacteria in the jejunum and their relationships with bile acids to influence cattle metabolism. Since the influences may start from the early age of cattle and the jejunum is not accessible in young animals, we first examined the bacterial communities in the rectum to obtain an initial impression of the influence of the two different diets. After the reach of market weight, jejunal digesta were collected for exploration. In addition to the analyses of the fecal and jejunal bacterial communities, we also examined whether the components of bile acids were associated with the composition of the intestinal bacteria. We profiled the microbial structure in jejunum by using the 16S rRNA amplicon sequencing, together with bile acid quantification, to characterize the influences of different diets on the jejunal bacteria.

## Results

### Fecal bacterial communities influenced by diets in young beef cattle

**Fig. 1:**
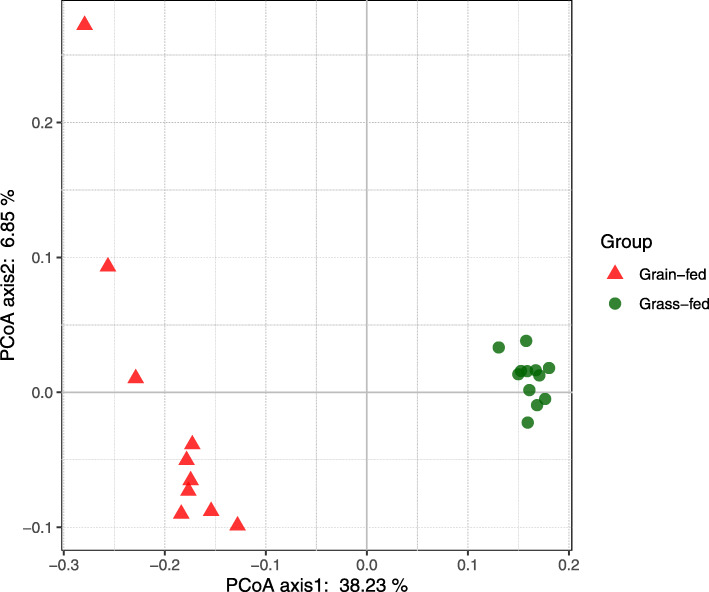
The effect of the diet on beta diversity of fecal bacterial community as shown by principal coordinate analysis (PCoA) based on unweighted Unifrac distance matrix

Based on the OTU table analyzed with Quantitative Insights Into Microbial Ecology (QIIME) [18], there were 19 microbial phyla identified from grass-fed and grain-fed groups (Additional file 1: Figure S1). The most abundant phylum was *Firmicutes*, ranging from 38.36 to 68.42% of relative abundance percentages, followed by *Bacteroidetes* (37.77%), *Proteobacteria* (3.96%), and *Verrucomicrobia* (1.20%). Diversity indices were examined for the fecal bacterial communities of grass-fed and grain-fed cattle. Alpha-diversity indices including Chao1, Shannon, Simpson, and Phylogenetic diversity (PD_whole_tree) were calculated and analyzed using the Wilcoxon rank-sum test between the two groups to determine the *p*-value for group comparisons (Table 1). Overall, the grass-fed group showed significantly higher alpha diversity indices than the grain-fed group (*p* <  0.05), suggesting that grass-feeding resulted in a more diverse gut bacteria communities than the grain-feeding. Ordination plots showed a distinct clustering pattern of global bacterial composition between grass-fed and grain-fed groups. For the 10 most abundant bacterial families between the two groups, such as *Ruminococcaceae* (10.92%), *Rikenellaceae* (6.20%), *Lachnospiraceae* (4.61%), and *Paraprevotellaceae* (4.34%), biplot of the principal component analysis (PCA) biplots showed a distinct separation of the two groups and also presented the associations of families with the two principle components as vectors (Additional file 1: Figure S2).The group separation was also observed on principal coordinate analysis (PCoA) (Fig. 1) and was further tested for significance by permutational multivariate analysis of variance (PERMANOVA). The influence of diets alone explained 37.24% of the variation in beta diversity in fecal bacterial communities (permutation *p* = 0.001).

**Table 1:** Alpha diversity indices in cattle fecal bacterial communities

Diversity Indices	Grain-fed	Grass-fed	***p***-value
Chao1	1596.03 ± 147.74	1876.16 ± 97.42	0.0001
Shannon	7.09 ± 0.21	7.86 ± 0.20	< 0.0001
Simpson	0.980 ± 0.006	0.990 ± 0.002	< 0.0001
PD_whole_tree	61.86 ± 3.77	72.25 ± 2.95	< 0.0001

### Differentially abundant taxa and important microbial features of fecal bacterial communities under different diets

**Fig. 2:**
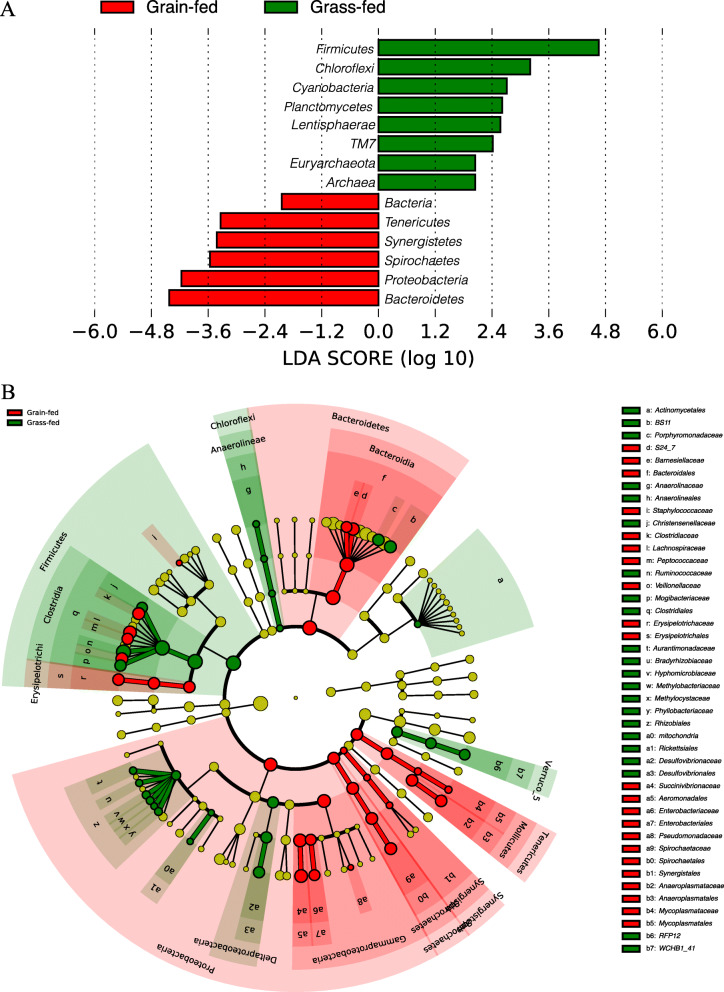
**a** Significantly discriminative taxa at a phylum level with absolute Linear Discriminant Analysis (LDA) scores ≥2.0 in fecal bacterial communities between grain-fed and grass-fed cattle. The bars represented the effect size (log LDA score) for a particular taxon in a group. **b** The cladogram representing the taxa in the fecal bacterial communities. Taxa at the family level that had significantly different abundances between grass-fed and grain-fed cattle with an absolute LDA score ≥ 2.0 were displayed

Differential taxa were examined by the Linear Discriminant Analysis (LDA) Effect Size (LEfSe) algorithm [19], fourteen phyla were identified to be differentially abundant (LDA score ≥ 2.0) (Fig. 2a). The negative score value of grain-fed group was because of the order of the numerator and denominator during effect size calculation. Forty-seven families showed significant differences in relative abundances between the two groups (absolute LDA score log10 ≥ 2.0). A cladogram was plotted at the family level with the notation of differential taxa under different diets in grass-fed and grain-fed groups (Fig. 2b). Among these differential families, *Ruminococcaceae, BS11,* and *Porphyromonadaceae* were the top three discriminative features in the grass-fed group, while *Succinivibrionaceae, S24–7, and Lachnospiraceae* were the top three discriminative families in the grain-fed group. At the OTU level, among the 4182 OTU detected, 402 had a significant difference in relative abundance (LDA ≥ 2.0). Of them, 144 OTU showed enrichment in the grain-fed group, and the other 258 OTU showed a higher abundance in the grass-fed group. The select significant OTU were listed in Additional file 1.

**Fig. 3:**
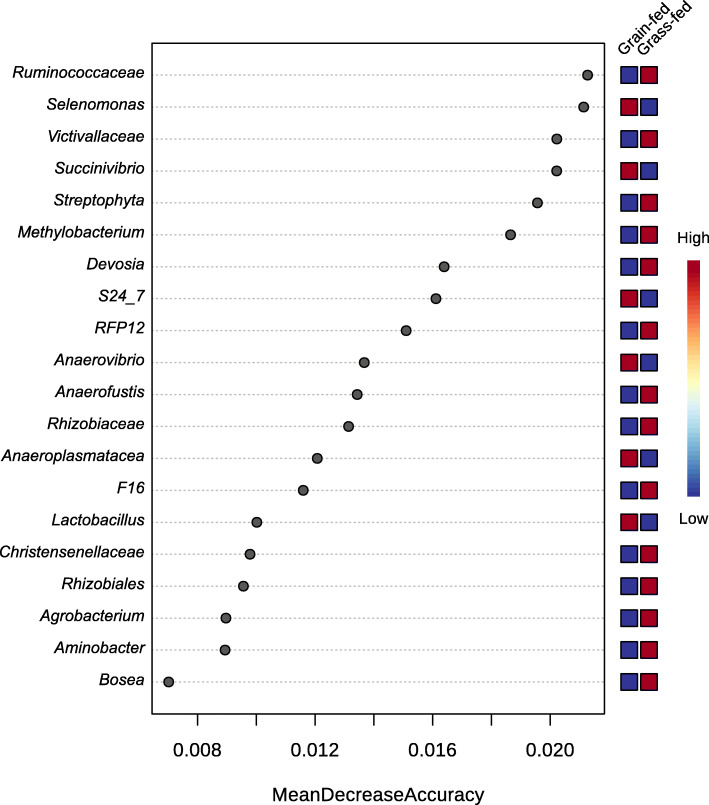
Random forest analysis on fecal bacterial communities of grass-fed and grain-fed Angus cattle. The y-axis, from top to bottom, displays the genera ranked by their relative importance based on Mean Decrease Accuracy in the classification of dietary groups

The abundance value based on the genus level was further evaluated by applying a random forest analysis for the group classification (m_try_ = 14, ntree = 500), and a 100% predictive accuracy in distinguishing the grass- from grain-fed groups was achieved (Fig. 3). The importance of features was measured by the decrease in mean accuracy. The 20 most important features were plotted, and their abundance levels were noted on the left side of the plot. These taxa held the highest discriminatory power between grass-fed and grain-fed groups and may serve as microbial biomarkers.

### The composition and structure of jejunal bacterial communities in beef cattle

A total of 24 phyla (Additional file 1), 44 classes, 77 orders, 149 families, and 263 genera were collectively detected. Of 24 phyla, the most abundant phylum was Firmicutes, which accounted for 63.11 to 98.21% in relative abundances. Other phyla with relatively high abundances included Proteobacteria (6.14%), Bacteroidetes (2.52%), Verrucomicrobia (1.92%), Actinobacteria (1.66%), and Elusimicrobia (0.89%). Among the 149 assigned families (Additional file 3), eight possessed a relative abundance higher than 1%, such as *Clostridiaceae* (33.82%), *Peptostreptococcaceae* (27.87%), *Ruminococcaceae* (6.03%), Enterobacteriaceae (5.69%), and *Lachnospiraceae* (5.62%). *Bacteroidaceae*, was also common in cattle, which accounted for approximately 0.99% abundance of jejunal bacteria families.

**Fig. 4:**
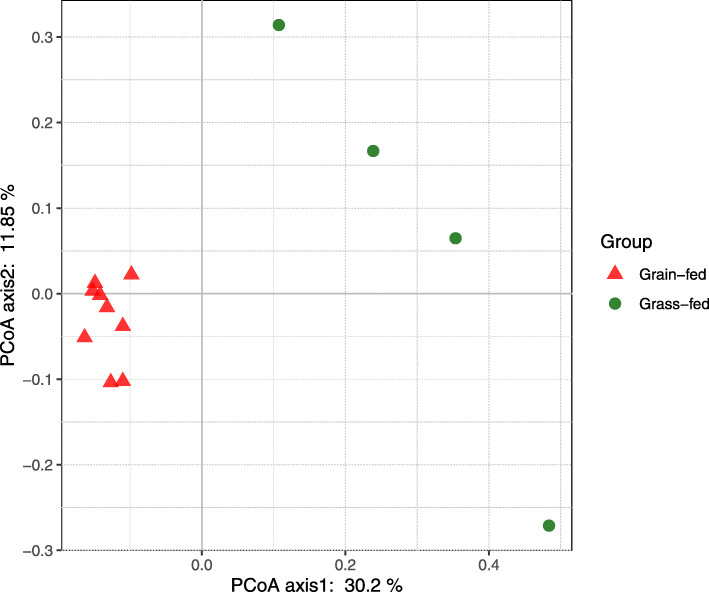
The dietary effect on beta diversity in the jejunal bacterial community as shown by principal coordinate analysis (PCoA) based on unweighted Unifrac distance matrix

Both alpha and beta diversities in the jejunal microbial community were also analyzed. Common alpha-diversity indices were further analyzed using a Wilcoxon rank sum test; and no significant differences in alpha diversity indices were detected between grass-fed and grain-fed groups (*p* > 0.05, Additional file 1: Table S2). Nevertheless, the grass-fed cattle tended to have a marginally higher alpha diversity than the grain-fed group. For example, the PD_whole_tree value was 66.77 ± 18.04 (mean ± SD) for the grass-fed group, comparing to 49.02 ± 9.46 in the grain-fed group (Additional file 1: Table S2). A rarefaction analysis based on Chao1 values suggested that the sequencing depth in the current study was sufficient (Additional file 1: Figure S3). As for the beta diversity analysis, PCA was plotted based on the relative abundance matrix of the top 10 families between the two groups; and a clear separation was observed (Additional file 1: Figure S5). PCoA based on unweighted Unifrac distance matrix also presented a distinct clustering pattern between grass-fed and grain-fed groups (Fig. 4). PERMANOVA results suggested that the influence of diets alone accounted for 27.55% of the variation in the jejunal bacterial communities in Angus beef cattle (permutation *p* = 0.002).

### The diet is the primary determinant of jejunal microbial composition

**Fig. 5:**
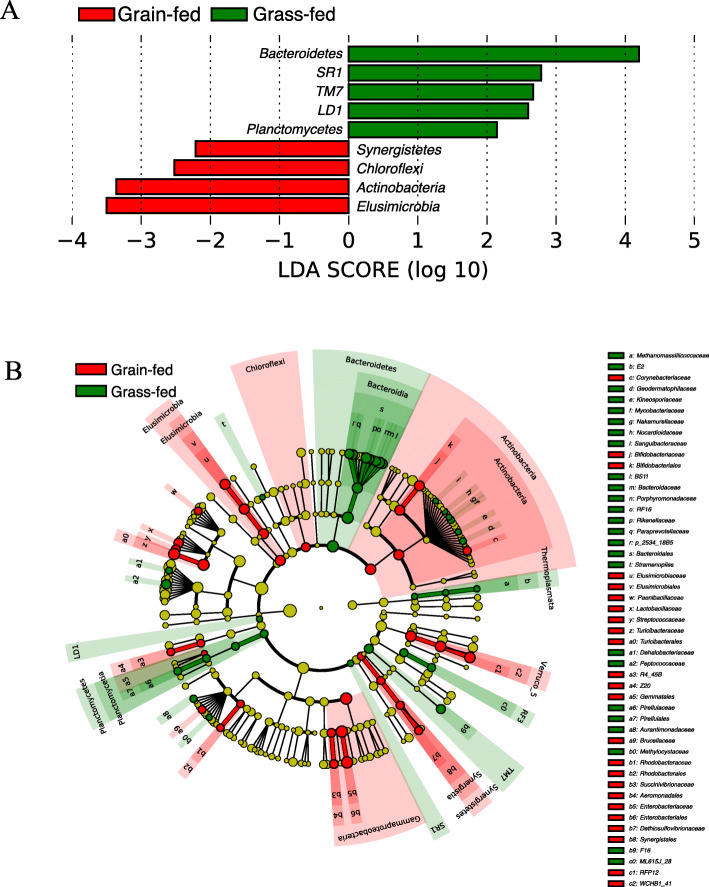
**a** Significantly discriminative phyla with LDA score ≥ 2.0 in jejunal bacterial communities between grain-fed and grass-fed cattle. **b** A cladogram representing the significant families in the jejunal bacterial communities. Only those taxa that had significant differences in relative abundance between grass-fed and grain-fed cattle with an absolute LDA score ≥ 2.0 were displayed

**Fig. 6:**
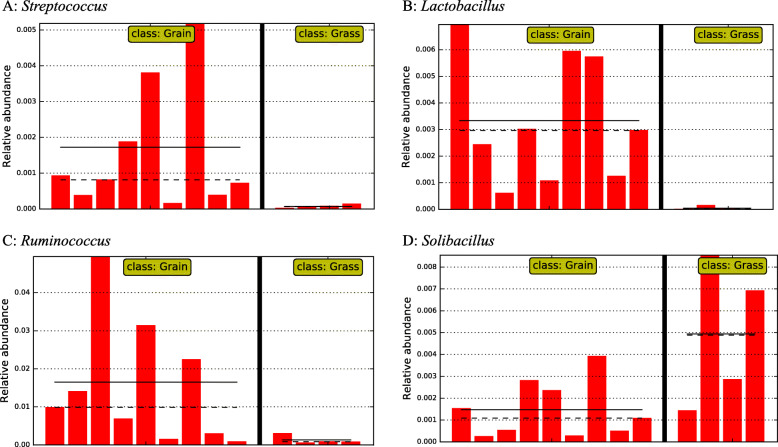
Selected genera displaying significant differences in relative abundance in jejunal bacterial communities between grass-fed and grain-fed cattle. **a** *Streptococcus*. **b** *Lactobacillus*. **c** *Ruminococcus*. **d** *Solibacillus*. X-axis represents individual animals in a group; y-axis represents relative abundance

Even though there was limited access to the external environment and low microbial abundance in the small intestine, diets still exerted several critical influences on microbial composition. Nine discriminative taxa at the phylum level were depicted (Fig. 5a). At the family level, 67 taxa showed significant differences in relative abundance between the two groups (LDA ≥ 2.0). For example, *Enterobacteriaceae, Turicibacteraceae, RFP12, Elusimicrobiaceae,* and *Bifidobacteriaceae* showed significantly higher abundance in the grain-fed group, whereas *Bacteroidaceae, Rikenellaceae, Paraprevotellaceae, BS11,* and *Nocardioidaceae* were significantly higher in abundance in the grass-fed group. A cladogram based on the family level was depicted (Fig. 5b), displaying taxa with significant differences in the jejunal bacteria. Forty-six named genera showed significant differences between the two groups (LDA ≥ 2.0, Additional file 3). For example, *Streptococcus*, *Lactobacillus*, and *Ruminococcus* were significantly higher in the jejunum of grain-fed group, whereas *Solibacillus* was significantly more abundant in the grass-fed group (Fig. 6). At the OTU level, 291 were significantly different in relative abundance between the grass-fed and grain-fed groups (Additional file 3). In comparison, 215 OTUs had higher relative abundance in the grass-fed group, and 76 OTUs showed higher relative abundance in the grain-fed group. Selected significantly different OTUs impacted by diets between the two groups were listed in Table 2.

**Table 2:** Select significantly different OTUs in jejunal bacterial communities between grain-fed and grass-fed cattle

Greengenes ID	Grain-fed	Grass-fed	LDA score	Annotation
347529	6.1592 ± 3.1357	0.7381 ± 0.4641	4.44	Bacteria|Firmicutes|Bacilli|Turicibacterales|Turicibacteraceae|Turicibacter
782953	5.8429 ± 7.3547	0.0838 ± 0.1517	4.46	Bacteria|Proteobacteria|Gammaproteobacteria|Enterobacteriales|Enterobacteriaceae
3829957	1.7923 ± 3.7084	0.0218 ± 0.0411	3.95	Bacteria|Proteobacteria|Gammaproteobacteria|Enterobacteriales|Enterobacteriaceae
533634	1.5224 ± 4.3245	0.0003 ± 0.0004	3.95	Bacteria|Firmicutes|Clostridia|Clostridiales|Lachnospiraceae|Moryella
302219	1.1924 ± 1.5725	0.0577 ± 0.0373	3.71	Bacteria|Verrucomicrobia|Verruco-5|WCHB1–41|RFP12
813277	1.0691 ± 1.2361	0.0001 ± 0.0002	3.69	Bacteria|Firmicutes|Clostridia|Clostridiales|Ruminococcaceae
23652	0.9982 ± 1.3779	0.0013 ± 0.0024	3.69	Bacteria|Elusimicrobia|Elusimicrobia|Elusimicrobiales|Elusimicrobiaceae
298198	0.8282 ± 0.5570	0.1283 ± 0.1665	3.52	Bacteria|Actinobacteria|Actinobacteria|Bifidobacteriales|Bifidobacteriaceae
579658	0.7178 ± 1.9300	0.0000 ± 0.0000	3.62	Bacteria|Firmicutes|Clostridia|Clostridiales|Lachnospiraceae|Moryella
3869719	0.6441 ± 0.5425	0.0750 ± 0.0743	3.44	Bacteria|Firmicutes|Clostridia|Clostridiales
570341	0.0001 ± 0.0002	0.8286 ± 1.6200	3.61	Bacteria|Firmicutes|Clostridia|Clostridiales
576472	0.0003 ± 0.0006	0.8228 ± 1.5401	3.59	Bacteria|Bacteroidetes|Bacteroidia|Bacteroidales|Bacteroidaceae|5-7 N15
347529	6.1592 ± 3.1357	0.7381 ± 0.4641	4.44	Bacteria|Firmicutes|Bacilli|Turicibacterales|Turicibacteraceae|Turicibacter
333300	0.1027 ± 0.1451	0.7123 ± 0.6684	3.48	Bacteria|Firmicutes|Clostridia|Clostridiales|Lachnospiraceae
586562	0.0002 ± 0.0006	0.6750 ± 1.3231	3.5	Bacteria|Firmicutes|Clostridia|Clostridiales|Ruminococcaceae
576712	0.0001 ± 0.0002	0.6492 ± 1.2683	3.47	Bacteria|Firmicutes|Clostridia|Clostridiales|Ruminococcaceae
592139	0.0000 ± 0.0000	0.5889 ± 1.1536	3.47	Bacteria|Firmicutes|Clostridia|Clostridiales|Ruminococcaceae
288681	0.0005 ± 0.0012	0.5337 ± 1.0051	3.44	Bacteria|Bacteroidetes|Bacteroidia|Bacteroidales|Bacteroidaceae
296290	0.0221 ± 0.0351	0.5000 ± 0.4341	3.36	Bacteria|Firmicutes|Clostridia|Clostridiales|Lachnospiraceae
309285	0.0000 ± 0.0000	0.3781 ± 0.7272	3.23	Bacteria|Firmicutes|Clostridia|Clostridiales

### Potential jejunal microbial pathways inferred from the 16S data

**Fig. 7:**
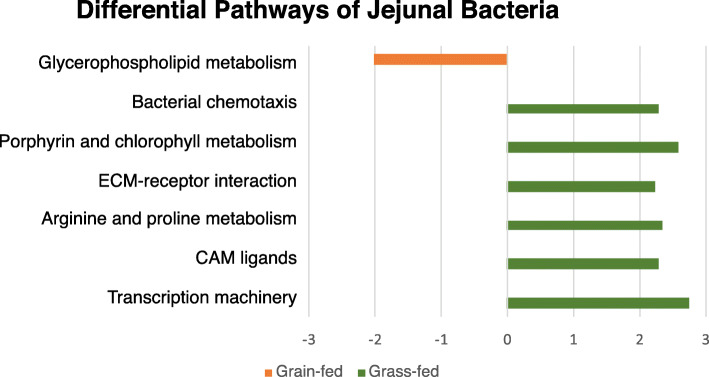
Predicted KEGG pathways significantly impacted by the diet in jejunal bacterial communities in Angus beef cattle (LDA score ≥ 2.0)

With Phylogenetic Investigation of Communities by Reconstruction of Unobserved States (PICRUSt) method [20], a total of 6909 Kyoto Encyclopedia of Genes and Genomes (KEGG) Orthology gene families were identified. Of them, five KEGG gene families showed significant differences in abundance between grass-fed and grain-fed groups based on the default LEfSe cutoff [19] (Additional file 3). Specifically, only one KEGG, insertion element IS1 protein InsB (K07480), was more abundant in the grain-fed group. In contrast, methyl-accepting chemotaxis protein (K03406), RNA polymerase sigma-70 factor, ECF subfamily (K03088), DNA topoisomerase III [EC:5.99.1.2] (K03169), and ABC-2 type transport system permease protein (K01992) had significantly higher abundance in the grass-fed group. In total, 328 KEGG pathways were identified. Some abundant functional pathways in the jejunal bacteria included membrane transport such as ABC transporters, genetic information processing such as DNA repair and recombination proteins, and nucleotide metabolisms. LEfSe analysis identified seven pathways that had significantly different abundance between grass-fed and grain-fed groups (Fig. 7).

### Associations between the jejunal bacteria and bile acids

Bile acids from gallbladder samples of eight cattle in each group were measured using a LC-MS/MS system. In total, 21 bile acids were identified and quantified, including both primary and secondary bile acids, and bile acid conjugates. Among them, 10 were significantly different between grass-fed and grain-fed groups (Table 3). Conjugated cholic acid and deoxycholic acid were detected at a relatively high concentration. For example, the levels of taurocholic acid, cholic and glycocholic acids were significantly higher in the grass-fed than the grain-fed group (Table 3; *p* <  0.05). Furthermore, at least six bile acids, including the conjugated form of common secondary bile acids, such as lithocholic acid and deoxycholic acid, were significantly higher in the grain-fed group. The other 11 detected bile acids, such as deoxycholic acid and ursodeoxycholic acid, were not significantly different between grass-fed and grain-fed groups (Additional file 1: Table S3), indicating their relatively low susceptibility to dietary influences in the gut of beef cattle. A critical concept of compositional balance has been introduced in recent studies for analyzing microbiome data [21, 22]. *Selbal* is a recently developed algorithm to identify the global microbial balance to find predictive microbial signatures of a phenotype of interest, specifically applicable on compositional data [22]. In our study, the predictive microbiome signatures that were most likely in association with secondary bile acids were obtained with *Selbal*. In the process, six secondary bile acids that were potentially related to bacterial bile acid conversion activities were used as the response variables for prediction in *Selbal*. Each time, one of the six bile acids was tested using the microbial abundance data at the genus level to perform modeling and variable selection. In total, twelve different taxa were selected among all the identified taxa, with some of them being used in more than one balances predictive of the corresponding bile acids (Table 4, Additional file 1: Figure S4).

**Table 3:** Ten bile acids with significant different levels between grass-fed and grain-fed cattle (concentration unit: μmol/ml)

Bile acids	Grain-fed	Grass-fed	***p***-value
Cholic acid^1^	0.884 ± 0.403	2.876 ± 2.45	0.0238
Glycocholic acid^1C^	171.912 ± 79.959	294.44 ± 63.734	0.0135
Glycodeoxycholic acid^2C^	57.035 ± 18.609	30.417 ± 8.094	0.0054
Glycolithocholic acid^2C^	1.001 ± 0.41	0.334 ± 0.17	0.0019
Glycoursodeoxycholic acid^2C^	0.073 ± 0.042	0.035 ± 0.013	0.0182
7-Ketodeoxycholic acid^2C^	0.08 ± 0.057	0.382 ± 0.425	0.0238
Taurocholic acid^1C^	206.139 ± 57.52	358.033 ± 46.647	0.0014
Taurodeoxycholic acid^2C^	79.243 ± 24.584	54.168 ± 11.158	0.0316
Taurolithocholic acid^2C^	1.075 ± 0.284	0.515 ± 0.08	0.0009
Total of tauroursodeoxycholic/taurohyodeoxycholic acid^2C^	0.152 ± 0.093	0.08 ± 0.016	0.0404

Bile acids were listed alphabetically, “1” denotes primary bile acids, “1C” denotes primary bile acids conjugates, “2C” denotes secondary bile acids conjugates

**Table 4:** Global balances in jejunal bacterial communities highly predictive for secondary bile acids

Bile acids	Numerator	Denominator	R-squared
Glycodeoxycholic_acid	f_Clostridiaceae|g_SMB53	f_Peptostreptococcaceae|g_Clostridium	0.895
Glycolithocholic_acid	f_Lachnospiraceae|g_Epulopiscium	f_Lactobacillaceae|g_Lactobacillus	0.863
		f_Staphylococcaceae|g_Staphylococcus	
Glycoursodeoxycholic_acid	f_Succinivibrionaceae|g_Succinivibrio	f_Veillonellaceae|g_Selenomonas	0.808
Taurodeoxycholic_acid	f_Enterococcaceae|g_Enterococcus	f_Turicibacteraceae|g_Turicibacter	0.825
Taurolithocholic_acid	f_Enterococcaceae|g_Enterococcus	o_Bacillales	0.92
	f_RFP12		
Total of tauroursodeoxycholic/taurohyodeoxycholic acid	f_Enterococcaceae|g_Enterococcus	f_Peptostreptococcaceae	0.773
	f_Peptostreptococcaceae|g_Clostridium		

“o,” “f,” and “g” denote taxonomic levels of order, family, and genus of the taxa, respectively. The balance (ratio) of the abundances of the bacteria in the numerator divided by the denominator works as a microbial signature that had a strong predictive power for the corresponding bile acids in this table

## Discussion

Grass- and grain-feeding represent two dominant feeding regimens in beef cattle production. Effects of diets on the gut bacteria are typically determined for ruminal and rectal microbial communities. However, microbial communities and their potential functions are critical for nutrient absorption in lower gut segments of cattle. In the jejunum, villi and microvilli increase the chance of food particles to encounter bile acids and digestive enzymes. Bacteria there actively participate in converting primary bile acids into secondary bile acids to aid digestion, especially the lipid [23]. In this study, we examined the fecal and jejunal bacteria communities and found that grass-fed and grain-fed methods altered the structure, diversity, and biological functional categories of the bacterial communities in both gut segments. Further, bile acids, influenced by diets, also exerted an influence on the jejunal bacteria communities.

From the feces of the 7-month-old young cattle under the two different diets, significant bacterial differences were evident from the phylum to the OTU level. The dominant phyla were in agreement with previous studies of cattle gut bacteria that Firmicutes was most abundant, followed by Bacteroidetes and Proteobacteria [8, 24]. At lower taxonomic ranks, *Ruminococcaceae*, the most abundant family, was higher in the grass-fed group, possibly because of its dependent on dietary fiber as an energy source [25]. *Ruminococcaceae* is also the most important family identified by random forest classification. Other genera also provided clues of possible influences of diets on fermentation strains in the upper forestomach. For example, *Succinivibrio* was 2-fold greater in abundance in the grain-fed group, possibly due to its abilities to ferment glucose into large amounts of succinic acid [26, 27]. These findings served as a baseline data and provided a reference for the jejunal bacteria characterization.

The unique environment and function of the small intestine make its bacterial community distinctive of those in the upper digestive tract [14]. From several previous studies, microbial diversities always decrease in this segment of cattle gut, because the sequential digestions in the upper gut and limited contact with the outside environment resulted in a reduced oxygen level [5, 9, 14, 24]. In our study, alpha diversity indices of jejunal bacterial communities did not achieve statistically significant differences between the two groups. Large variations among individuals, the unfavorable environment of the small intestine itself, and fast rate of digesta passage as well as relatively small sample size could potentially explain the phenomenon. Significant differences of specific taxa in bacterial communities of jejunum could have important health implications for beef cattle production. Lactate producers, such as *Lactobacillus*, *Streptococcus*, and *Bifidobacterium* [28, 29], and *Megasphaera*, a commonly known genus harboring many lactic acid utilizers [30], all showed higher abundances in grain-fed cattle. These genera have long been observed to be more abundant in high-grain diet and acidic rumen [29, 31]. *Solibacillus* was reported to decrease when diets were shifted to sub-acute ruminal acidosis induction diets, which potentially related to valerate metabolism [32]. Our study further showed that the same trend persisted from the rumen to the jejunum of grain-fed cattle. From the aspect of cattle health, these observations suggested that grain-feeding may lead to an acidic gut environment globally and had a significant impact not only in the rumen, but also in the jejunum.

As for functional categories of the bacterial communities in the jejunum, few KEGG gene families and potential pathways showed significant differences between the two feeding groups. In the grass-fed group, proteins involving microbial genetic activities were higher, which could potentially explain the enriched transcription machinery pathway. Porphyrin and chlorophyll metabolism were also higher, likely because of the higher intake of plant contents to the gut, since prophyrin is a structural compound of the chlorophyll. As for the grain-fed group, glycerophospholipid metabolism showed a significant increase. Glycerophospholipids were major complex lipids composed of glycerol, two fatty acids, phosphate, and an amino alcohol [33]. Higher lipids in grain-fed regimen, with the digestion facilitated by bile acids, could provide higher levels of glycerol and fatty acids in the small intestine for nutrient absorption, triggering more activities of glycerophospholipids metabolisms of microbes in grain-fed cattle jejunum. Further explorations will likely facilitate our understanding of the mechanisms on how these gut bacterial pathways were regulated under various diets.

Bacterial communities of the small intestine play a critical role in bile acid conversion. It has been reported that, gram-positive bacteria in the order of *Clostridiales*, including *Ruminococcaceae,* can produce 7α-dehydroxylation to convert primary bile acids into secondary bile acids [34]. In this study, six identified signatures, belonging to the family *Ruminococcaceae*, were more abundant in the grain-fed group, which might contribute to the observed higher secondary bile acids. Also, the increase of *Lactobacillus, Clostridium,* and *Bifidobacterium* in the grain-fed group can aid to deconjugate the amide bond between bile acids with glycine or taurine, to make bile acids available as substrates for further biotransformation by other microbes [34]. These differential taxa and microbial signatures provided evidence that there existed novel associations between gut bacteria and elevated secondary bile acids in the grain-fed cattle. In addition, potentially less healthy fatty acid profiles in grain-fed beef are worthy of further investigation.

## Conclusions

In ruminants, a comprehensive survey of microbial composition in the small intestine digesta of grain- and grass-fed beef cattle is still in its infancy. In this study, bacterial profiles and candidate microbial biomarkers were identified in fecal matters of 7-month-old Angus cattle, suggesting an early influence of diet on the bacterial communities in beef cattle. Bacteria in the jejunum are much less diverse where Firmicutes is most predominant and accounts for more than 90% relative abundance, which may result from the lower pH and a higher rate of digesta passage. Moreover, differences in various microbial diversity indices there between the grass-fed and grain-fed cattle are not significant, while the microbial composition and pathways differ significantly. Most importantly, the gut bacteria in association with bile acids and microbial signatures are identified. There is an increased level of secondary bile acids in the grain-fed group, as well as increased bacterial taxa that promote the conversion of bile acids. Moreover, we identified bacterial signatures that are strongly associated with secondary bile acids. Furthermore, a microbial metabolic pathway, glycerophospholipid metabolism, is also enriched in the grain-fed group. Our results may provide important clues about the bacterial communities in the lower GI tract of beef cattle and can facilitate the comprehensive understanding of the jejunal microbes and their roles in bile acid metabolism.

## Methods

### UMD Wye Angus herd and experimental design

Steers selected for this study came from a population of cattle in the University of Maryland, Wye Angus beef cattle herd. This herd has been kept closed for over 50 years and maintained a very similar genetic background for all offspring, which reduces the genetic variations of groups for our study. Two kinds of feeding methods, grain-fed and free-range grass-fed methods, were used in this herd as two experimental groups in this study. The grain-fed group received finishing diets including corn silage, shelled corn, soybean, and trace minerals, which were high in energy and protein. The grass-fed group had free access to grazed alfalfa and consumed bailage during cold seasons. The hay contained no fertilizers, pesticides, or other artificial chemicals. Steers from the grass-fed group in this herd were not fed with any animal, agricultural, or industrial byproducts, and were not supplied with any types of grain. Each steer’s date of birth, birth weight, dam, and sire information were all recorded. Every 24 to 28 days, body weight was measured, and average daily gain (ADG) was calculated. All metadata of the steers were recorded.

### Sample collection of fecal and jejunal contents

Twenty-two young male cattle were obtained and raised at UMD Wye Angus herd. Twelve animals were fed with grass ad libitum. The other 10 cattle were fed with grain diets. Around 7 months old, feces at the rectum of both groups of cattle were sampled at UMD Wye Angus farm (Queenstown, MD) and immediately frozen in dry ice and stored at − 80 °C freezer in the lab until microbial DNA extraction. After the young animals under two dietary systems reached their marketing weight, cattle were slaughtered and jejunal contents were immediately collected for microbial DNA extraction.

### DNA extraction, 16 s rRNA gene amplification, and sequencing

Microbial DNA from feces and jejunal contents was extracted using QIAamp DNA stool kit (Qiagen, Valencia, CA) with one protocol modification that replaced the lysis procedure in protocol with an eight-minute 95 °C incubation in a water bath. DNA concentration was then measured using NanoDrop 2000 spectrophotometer (Thermo Fisher Scientific, Waltham, MA USA). 16S rRNA gene sequencing was performed as previously described [35]. Briefly, QIAamp DNA stool kit (Qiagen, Valencia, CA) was used for microbial DNA extraction for both fecal and jejunal contents. DNA concentration was then measured using NanoDrop 2000 spectrophotometer (Thermo Fisher Scientific, Waltham, MA USA). Hypervariable V3-V4 regions of the 16S rRNA gene were amplified through PCR from 20 ng of total DNA with PAGE-purified Illumina platform-compatible adaptor oligos. The primer sequences were as follows: forward primer, 341/357F, CCTACGGGNGGCWGCAG; reverse primer, 805/785R: GACTACHVGGGTATCTAATCC. DNA concentration and sizes were quantified using a BioAnalyzer 2000 (Agilent, Palo Alto, CA). Amplicons were finally purified using Agencourt AMPure XP bead kits (Beckman Coulter Genomics, Danvers, MA). The library pool was sequenced in an Illumina MiSeq sequencer with an Illumina MiSeq Reagent Kit according to a protocol previously described [36].

### Bile acids measurements

Bile acid composition was measured from the liquid bile by a commercial lab (Creative Proteomics, Shirley, NY) according to a procedure published previously [37]. Five microliter of cattle bile was mixed with 995 μL of methanol in a microfuge tube. After mixing by vortex for 15 s and sonication for 2 min, the separation was done by centrifuging at 15,000 rpm at 10 °C for 15 min. Eight microliter of the supernatant was then mixed with 992 μL of 50% methanol. After preprocessing, 20 μl aliquots were used for quantitation by UPLC-(−)ESI-MRM/MS according to a procedure published previously [37]. Standard curves for bile acid concentrations were performed, as follows: a STD mix containing standard substances of all the quantified bile acids was dissolved in 50% methanol, and this standard solution was used as S1. The S1 standard solution was diluted in a series at the same dilution ratio of 1 to 4 with 50% methanol to have standard solutions of S2 to S10. Fifty microliter of each of S1 to S10 was mixed with 50 μl of 14 D4-bile acid SIS mix (as internal standards) and 100 μl of 50% methanol. Twenty microliter aliquots were injected to record the data files so as to prepare calibration curves. Linear regression (As/Ai peak area ratio versus concentration) was used for calculation of bile acid concentrations measured in the individual samples. The ratio of the peak areas of the sample (As) to the peak area of the internal standard (Ai) were measured to compare with the concentration ratio derived for each calibration standard to calculate the Response Factor (RF). SciexMultiQuant was used for data processing. Wilcoxon rank sum test was used to compare the difference of bile acid from grass-fed to grain-fed group with a selected threshold for *p*-values < 0.05.

### Bioinformatics and data analysis

Raw sequences were first analyzed with FastQC (version 0.11.5) to examine the quality of sequencing. The first four maximally degenerate bases (“NNNN”) at the most 5′ end, designed to maximize the diversity during sequencing run to better identify unique clusters and improve base-calling accuracy, were trimmed with Trimmomatic version 0.38 [38]. Then, the processed pair-end reads were merged using PandaSeq v2.11 with default parameters to join the reads into representative complete nucleotide sequences (contigs) [39]. Overlapped reads with high mismatches and low-quality scores were filtered and removed during this process. Bacterial communities of feces and jejunum were examined based on the OTU table generated from the closed reference pipeline of Quantitative Insights Into Microbial Ecology (QIIME) version 1.9.1 [18, 40]. Operational taxonomic unit (OTU) was identified using a closed-reference OTU picking protocol with a threshold of 97% similarity. Taxonomic assignment was performed based on the GreenGenes database v13.8 [41]. Alpha-diversity was obtained at OTU level with *alpha_diversity.py* in QIIME and analyzed using a Wilcoxon rank sum test in R. For beta-diversity, *beta_diversity.py* in QIIME was used to obtain distance matrices. PCA on phylum level abundance, PCoA based on unweighted Unifrac distance followed by PERMANOVA, were performed with vegan [42] and factoextra [43] packages of R to examine the community level variances. The graphs were plotted with ggplot2 [44]. PICRUSt (v1.1.2) was used with default parameters to predict metagenomics, KEGGs gene families with regarding contributions of OTU, and functional categories between grass-fed and grain-fed groups based on 16S rRNA marker gene sequences. PICRUSt *normalize_by_copy_number.py* was used for normalization before functional pathway prediction. This step divided each OTU’s abundance value of each sample by its predicted 16S rRNA copy number based on Greengenes to correct the OTU table abundances [20]. The software takes the OTU table from QIIME as input. After reads normalization with *normalize_by_copy_number.py*, the workflow *predict_metagenomes.py* was applied to predict metagenomes and obtain KEGG Orthologs. Predicted metagenome functions were finally analyzed by *categorize_by_function.py* to collapse thousands of KEGG Orthologs into higher functional categories (pathways). LEfSe algorithm [19] was used in the differential analysis to identify significantly different taxa, KEGGs, and pathways between the grass-fed group and grain-fed groups. LEfSe can identify abundant differences of representative features between two or more sample conditions, considering samples’ biological categories as well as statistical significance to perform comparative analysis based on feature abundance. Non-parametric factorial Kruskal-Wallis (KW) sum-rank test, Wilcoxon rank-sum test, and a Linear Discriminant Analysis were used together to identify differential features and effect sizes. The absolute values of this effect size could be interpreted as the scale of difference between the comparison groups [19]. In addition, random forest (RF) classification was performed with the group as the class using R randomForest package v4.6–14 [45]. The number of trees (ntree) in the forest was set to 501 and the number of features randomly sampled for each split of the tree (m_try_) was 14. The out-of-bag decrease in accuracy was averaged in all trees for a variable (mean decrease accuracy) and used as the measurement of variable importance. *Selbal* was used to identify global balances of the two groups and identify microbial signatures predictive of significantly different secondary bile acids with default parameters [22].

## Supplementary information

**Additional file 1: Figure S1.** The relative abundance of fecal bacterial communities at a phylum level. **Figure S2.** Ordination biplots of principal component analysis (PCA) in fecal bacterial communities. **Table S1.** Selected OTUs. **Figure S3.** The relative abundance of jejunal bacteria at a phylum level. **Table S2.** Alpha diversity indices of bacterial communities in the jejunum. **Figure S4.** Rarefaction curves of the jejunal bacterial communities. **Figure S5.** Ordination biplots of PCA on the jejunal bacteria. **Table S3.** Bile acids in the jejunum, including those without significant differences in concentrations between two dietary groups. **Figure S6.** The global balances predictive for the levels of secondary bile acids.

**Additional file 2:.** OTU table and the family level abundance table for fecal bacterial communities.

**Additional file 3:.** Feature tables, including OTU table, family level abundance table, and the differential genera, OTUs, and KEGGs.

## Data Availability

The 16S rRNA gene sequencing data are available in the NCBI Sequence Read Archive (SRA) under Bioproject PRJNA608270.

## Abbreviations

ADG: Average daily gain
DCA: Deoxycholic acid
DNA: Deoxyribonucleic acid
GI tract: Gastrointestinal tract
KEGG: Kyoto Encyclopedia of Genes and Genomes
LCA: Lithocholic acid
LC-MS: Liquid chromatography–mass spectrometry
LEfSe: Linear Discriminant Analysis (LDA) Effect Size
MAES: Maryland Agricultural Experiment Station
OTU: Operational taxonomic unit
PCA: Principal component analysis
PCR: Polymerase chain reaction
PCoA: Principal coordinate analysis
PD: Phylogenetic diversity
PERMANOVA: Permutational multivariate analysis of variance
PICRUSt: Phylogenetic Investigation of Communities by Reconstruction of Unobserved States
QIIME: Quantitative Insights Into Microbial Ecology
RF: Response Factor
SRA: Sequence Read Archive
UMCP-IACUC: Institutional Animal Care and Use Committee at the University of Maryland
UMD: University of Maryland

## References

[1] Xue H, Mainville D, You W, Nayga RM (2010). Consumer preferences and willingness to pay for grass-fed beef: empirical evidence from in-store experiments. Food Qual Prefer.

[2] Thomas HS, Black B (2018). Storey's guide to raising beef cattle : health, handling, breeding.

[3] Muegge BD, Kuczynski J, Knights D, Clemente JC, González A, Fontana L (2011). Diet drives convergence in gut microbiome functions across mammalian phylogeny and within humans. Science.

[4] Van Soest PJ (1994). Nutritional ecology of the ruminant.

[5] Mao S, Zhang M, Liu J, Zhu WJ (2015). Characterising the bacterial microbiota across the gastrointestinal tracts of dairy cattle: membership and potential function. Sci Rep.

[6] Wang J, Fan H, Han Y, Zhao J, Zhou Z (2017). Characterization of the microbial communities along the gastrointestinal tract of sheep by 454 pyrosequencing analysis. Asian Australas J Anim Sci.

[7] Khafipour E, Li S, Tun H, Derakhshani H, Moossavi S, Plaizier JJAF (2016). Effects of grain feeding on microbiota in the digestive tract of cattle. Anim Front.

[8] Myer P, Freetly H, Wells J, Smith T, Kuehn LJJ (2017). Analysis of the gut bacterial communities in beef cattle and their association with feed intake, growth, and efficiency. J Anim Sci.

[9] Myer PR, Wells JE, Smith TP, Kuehn LA, Freetly HC (2016). Microbial community profiles of the jejunum from steers differing in feed efficiency. J Anim Sci.

[10] Deusch S, Tilocca B, Camarinha-Silva A, Seifert J (2015). News in livestock research—use of Omics-technologies to study the microbiota in the gastrointestinal tract of farm animals. Comput Struct Biotechnol J.

[11] Kamada N, Chen GY, Inohara N, Núñez G (2013). Control of pathogens and pathobionts by the gut microbiota. Nat Immunol.

[12] Sheriha GM, Waller GR, Chan T, Tillman ADJL (1968). Composition of bile acids in ruminants. Lipids.

[13] Bauchart D (1993). Lipid absorption and transport in ruminants. J Diary Sci.

[14] Durso LM, Miller DN, Schmidt TB, Callaway T (2017). Tracking bacteria through the entire gastrointestinal tract of a beef steer. Agric Environ Lett.

[15] Lingle JB, Koch CR, Barao SM (2001). The breed of noble bloods.

[16] Carrillo JA, He Y, Li Y, Liu J, Erdman RA, Sonstegard TS (2016). Integrated metabolomic and transcriptome analyses reveal finishing forage affects metabolic pathways related to beef quality and animal welfare. Sci Rep.

[17] Li Y, Carrillo JA, Ding Y, He Y, Zhao C, Liu J (2015). Transcriptomic profiling of spleen in grass-fed and grain-fed angus cattle. PLoS One.

[18] Caporaso JG, Kuczynski J, Stombaugh J, Bittinger K, Bushman FD, Costello EK (2010). QIIME allows analysis of high-throughput community sequencing data. Nat Methods.

[19] Segata N, Izard J, Waldron L, Gevers D, Miropolsky L, Garrett WS (2011). Metagenomic biomarker discovery and explanation. Genome Biol.

[20] Langille MG, Zaneveld J, Caporaso JG, McDonald D, Knights D, Reyes JA (2013). Predictive functional profiling of microbial communities using 16S rRNA marker gene sequences. Nat Biotechnol.

[21] Hensley-McBain T, Wu MC, Manuzak JA, Cheu RK, Gustin A, Driscoll CB (2019). Increased mucosal neutrophil survival is associated with altered microbiota in HIV infection. PLoS Pathog.

[22] Rivera-Pinto J, Egozcue J, Pawlowsky-Glahn V, Paredes R, Noguera-Julian M, Calle M (2018). Balances: a new perspective for microbiome analysis. MSystems..

[23] Nickel R, Schummer A, Seiferle E, Sack WO (1979). The viscera of the domestic mammals.

[24] de Oliveira MNV, Jewell KA, Freitas FS, Benjamin LA, Tótola MR, Borges AC (2013). Characterizing the microbiota across the gastrointestinal tract of a Brazilian Nelore steer. Vet Microbiol.

[25] Jensen BB, Jørgensen H (1994). Effect of dietary fiber on microbial activity and microbial gas production in various regions of the gastrointestinal tract of pigs. Appl Environ Microbiol.

[26] Bryant MP, Small N (1956). Characteristics of two new genera of anaerobic curved rods isolated from the rumen of cattle. J Bacteriol.

[27] Lau SKP, Teng JLL, Chiu TH, Chan E, Tsang AKL, Panagiotou G (2018). Differential microbial communities of omnivorous and herbivorous cattle in Southern China. Comput Struct Biotechnol J.

[28] Yang HE, Zotti CA, McKinnon JJ, McAllister TA (2018). Lactobacilli are prominent members of the microbiota involved in the ruminal digestion of barley and corn. Front Microbiol.

[29] Goad D, Goad C, Nagaraja TJ (1998). Ruminal microbial and fermentative changes associated with experimentally induced subacute acidosis in steers. J Anim Sci.

[30] Kung L, Hession AO (1995). Preventing in vitro lactate accumulation in ruminal fermentations by inoculation with Megasphaera elsdenii. J Anim Sci.

[31] Silva L, Pereira O, Silva T, Leandro E, Paula R, Santos S (2018). Effects of Lactobacillus buchneri isolated from tropical maize silage on fermentation and aerobic stability of maize and sugarcane silages. Grass Forage Sci.

[32] Mao S, Zhang R, Wang D, Zhu W (2012). The diversity of the fecal bacterial community and its relationship with the concentration of volatile fatty acids in the feces during subacute rumen acidosis in dairy cows. BMC Vet Res.

[33] Tocher DR (1995). Glycerophospholipid metabolism. Biochemistry and molecular biology of fishes.

[34] Urdaneta V, Casadesús J (2017). Interactions between bacteria and bile salts in the gastrointestinal and hepatobiliary tracts. Front Med.

[35] Li RW, Li W, Sun J, Yu P, Baldwin RL, Urban JF (2016). The effect of helminth infection on the microbial composition and structure of the caprine abomasal microbiome. Sci Rep.

[36] Liu F, Smith AD, Solano-Aguilar G, Wang TTY, Pham Q, Beshah E (2020). Mechanistic insights into the attenuation of intestinal inflammation and modulation of the gut microbiome by krill oil using in vitro and in vivo models. Microbiome..

[37] Han J, Liu Y, Wang R, Yang J, Ling V, Borchers CH (2015). Metabolic profiling of bile acids in human and mouse blood by LC–MS/MS in combination with phospholipid-depletion solid-phase extraction. Anal Chem.

[38] Bolger AM, Lohse M, Usadel B (2014). Trimmomatic: a flexible trimmer for Illumina sequence data. Bioinformatics.

[39] Masella AP, Bartram AK, Truszkowski JM, Brown DG, Neufeld JD (2012). PANDAseq: paired-end assembler for illumina sequences. BMC Bioinformatics.

[40] Navas-Molina JA, Peralta-Sánchez JM, González A, McMurdie PJ, Vázquez-Baeza Y, Xu Z (2013). Advancing our understanding of the human microbiome using QIIME. Methods Enzymol.

[41] DeSantis TZ, Hugenholtz P, Larsen N, Rojas M, Brodie EL, Keller K (2006). Greengenes, a chimera-checked 16S rRNA gene database and workbench compatible with ARB. Appl Environ Microbiol.

[42] Oksanen J, Kindt R, Legendre P, O’Hara B. The vegan package.

[43] Kassambara A, Mundt F. Factoextra: extract and visualize the results of multivariate data analyses. 2016;1(3):50–4.

[44] Wickham H (2016). ggplot2: elegant graphics for data analysis.

[45] Liaw A, Wiener M. Classification and regression by randomForest. R news. 2002;2(3):18–22.

