# Supplementary material for: Diet-induced changes in bacterial communities in the jejunum and their associations with bile acids in Angus beef cattle
Source: Anim Microbiome. 2020 Sep 16;2:33. doi: 10.1186/s42523-020-00051-7 (PMC7807434; doi:10.1186/s42523-020-00051-7)
Supplement: Supplementary file 1 — Additional file 1: Figure S1. The relative abundance of fecal bacterial communities at a phylum level. Figure S2. Ordination biplots of principal component analysis (PCA) in fecal bacterial communities. Table S1. Selected OTUs. Figure S3. The relative abundance of jejunal bacteria at a phylum level. Table S2. Alpha diversity indices of bacterial communities in the jejunum. Figure S4. Rarefaction curves of the jejunal bacterial communities. Figure S5. Ordination biplots of PCA on the jejunal bacteria. Table S3. Bile acids in the jejunum, including those without significant differences in concentrations between two dietary groups. Figure S6. The global balances predictive for the levels of secondary bile acids. [file 42523_2020_51_MOESM1_ESM.pdf]

Additional file 1

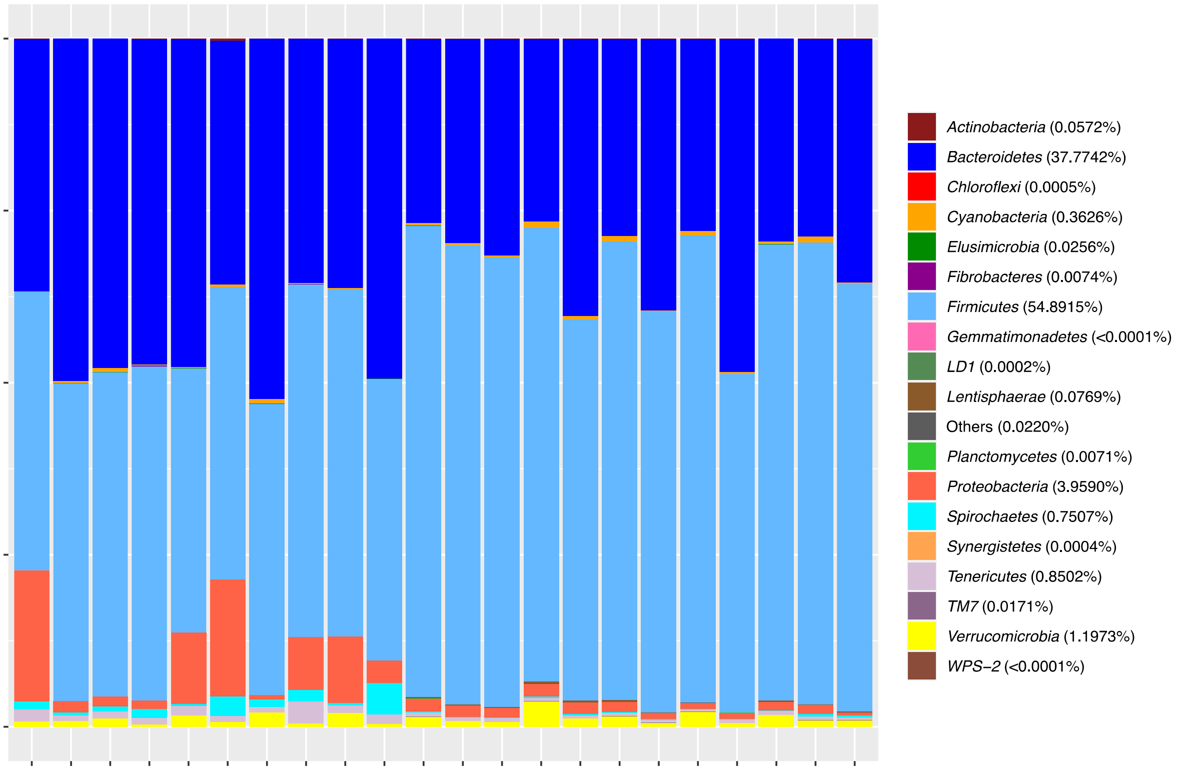

Figure S1 The relative abundance of fecal bacterial communities at a phylum level.

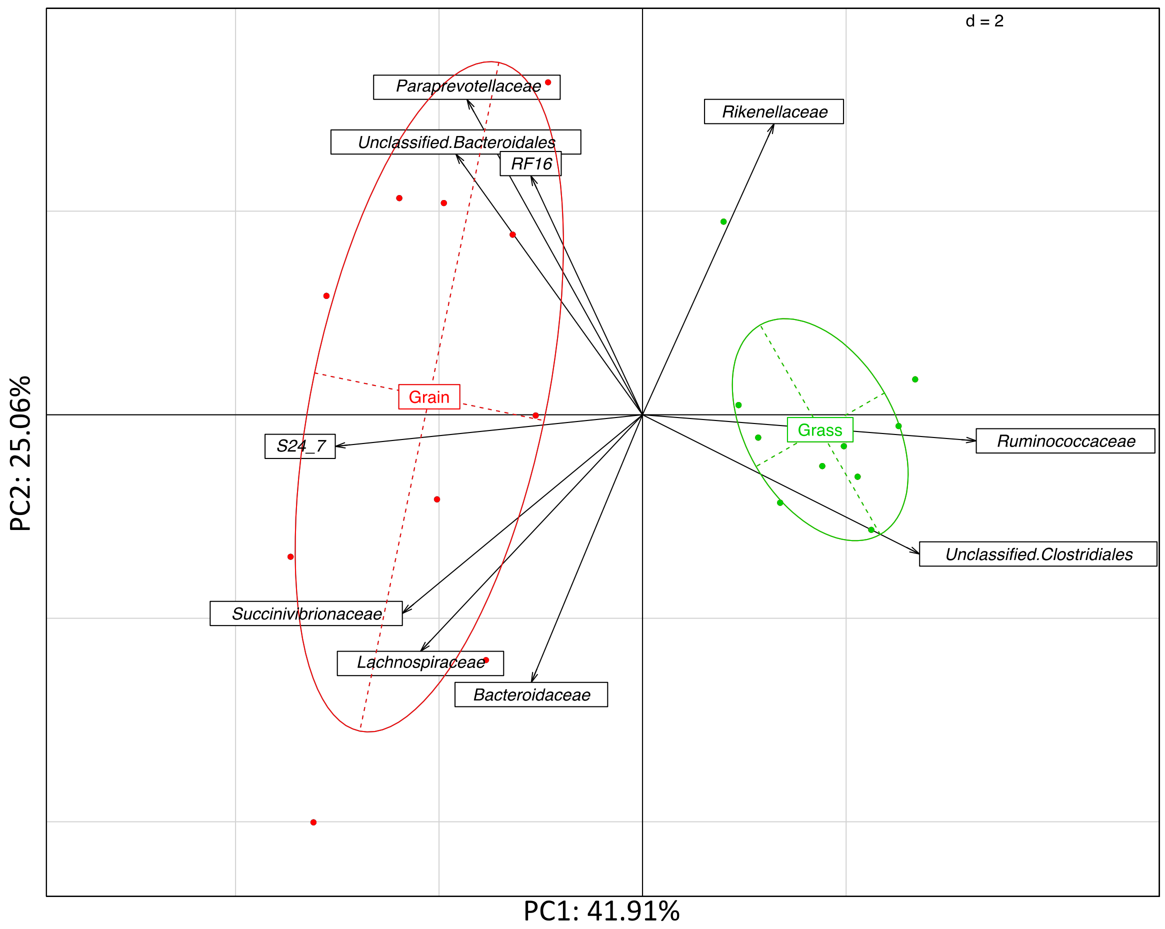

Figure S2 Ordination biplots of principal component analysis (PCA) in fecal bacterial communities. Arrows display the directions and relative importance of the top 10 abundant families. The variability in the fecal bacterial communities is explained on x-axis with 41.91% and on y-axis with 25.06%. "d" on the top right of the figure stands for "dimension".

Table S1 Selected OTUs in relative abundance in the fecal microbiome of grain-fed and grass-fed Angus beef cattle. The OTU was identified based on Greengenes 16S rRNA database version 13_8. The numbers denoted the relative abundance (Mean ± SD) of corresponding OTUs in the grain-fed and grass-fed group. The LDA log10 score was the calculated logarithm of LDA scores using the LefSe algorithm.

**GreenGenes ID**	**Grain-fed**	**Grass-fed**	**LDA log10 score**	**Annotation (Domain|Phylum|Class|Order|Family|Genus|Species)**
327184	0.0077 ± 0.0043	0.0319 ± 0.0069	4.08	Bacteria|Firmicutes|Clostridia|Clostridiales
509709	0.0078 ± 0.0039	0.0289 ± 0.0069	4.03	Bacteria|Firmicutes|Clostridia|Clostridiales|Lachnospiraceae
199226	0.0103 ± 0.0066	0.0213 ± 0.0092	3.78	Bacteria|Firmicutes|Clostridia|Clostridiales|Ruminococcaceae
298717	0.0178 ± 0.0123	0.0115 ± 0.0055	3.44	Bacteria|Firmicutes|Clostridia|Clostridiales|Ruminococcaceae
330414	0.0253 ± 0.0340	0.0000 ± 0.0000	4.08	Bacteria|Firmicutes|Clostridia|Clostridiales
1112614	0.0203 ± 0.0208	0.0038 ± 0.0008	3.92	Bacteria|Firmicutes|Clostridia|Clostridiales|Ruminococcaceae
85199	0.0172 ± 0.0098	0.0060 ± 0.0020	3.74	Bacteria|Firmicutes|Clostridia|Clostridiales|Veillonellaceae
146314	0.0233 ± 0.0409	0.0000 ± 0.0000	4.05	Bacteria|Tenericutes|Mollicutes|RF39
341737	0.0125 ± 0.0048	0.0075 ± 0.0026	3.33	Bacteria|Firmicutes|Clostridia|Clostridiales
177109	0.0069 ± 0.0041	0.0110 ± 0.0031	3.28	Bacteria|Firmicutes|Clostridia|Clostridiales|Ruminococcaceae|Oscillospira
593357	0.0062 ± 0.0019	0.0114 ± 0.0060	3.38	Bacteria|Bacteroidetes|Bacteroidia|Bacteroidales|Prevotellaceae|Prevotella
323737	0.0111 ± 0.0061	0.0044 ± 0.0014	3.53	Bacteria|Firmicutes|Clostridia|Clostridiales|Ruminococcaceae
248126	0.0160 ± 0.0144	0.0000 ± 0.0000	3.90	Bacteria|Firmicutes|Clostridia|Clostridiales|Peptostreptococcaceae
554560	0.0115 ± 0.0061	0.0036 ± 0.0014	3.60	Bacteria|Firmicutes|Clostridia|Clostridiales|Ruminococcaceae
287909	0.0004 ± 0.0010	0.0127 ± 0.0076	3.79	Bacteria|Firmicutes|Clostridia|Clostridiales
287798	0.0083 ± 0.0032	0.0056 ± 0.0016	3.12	Bacteria|Firmicutes|Erysipelotrichi|Erysipelotrichales|Erysipelotrichaceae
341730	0.0091 ± 0.0037	0.0049 ± 0.0015	3.32	Bacteria|Firmicutes|Clostridia|Clostridiales|Lachnospiraceae|Coprococcus
332778	0.0036 ± 0.0014	0.0093 ± 0.0022	3.45	Bacteria|Firmicutes|Clostridia|Clostridiales|Lachnospiraceae
2051	0.0125 ± 0.0098	0.0016 ± 0.0016	3.76	Bacteria|Bacteroidetes|Bacteroidia|Bacteroidales|Prevotellaceae|Prevotella
353691	0.0073 ± 0.0031	0.0057 ± 0.0007	2.80	Bacteria|Firmicutes|Erysipelotrichi|Erysipelotrichales|Erysipelotrichaceae

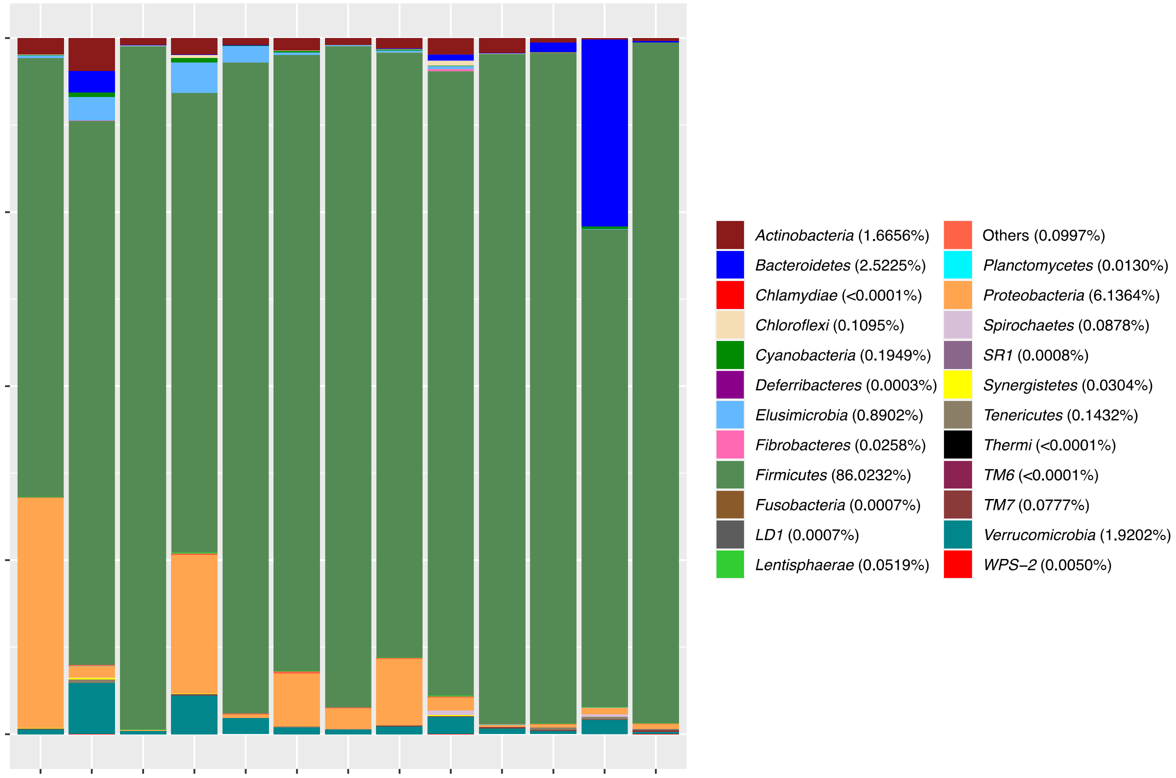

Figure S3 The relative abundance of jejunal bacteria at a phylum level.

Table S2 Alpha diversity indices of bacterial communities in the jejunum. Wilcoxon rank sum test was used to calculate p-values.

Diversity Indices	Grain-fed	Grass-fed	p-value
Chao1	1225.62 ± 468.55	1536.97 ± 706.42	0.1535
Shannon	4.07 ± 0.95	4.69 ± 2.40	0.9333
Simpson	0.83 ± 0.08	0.990 ± 0.11	0.9333
PD_whole_tree	49.02 ± 9.46	66.77 ± 18.04	0.1091

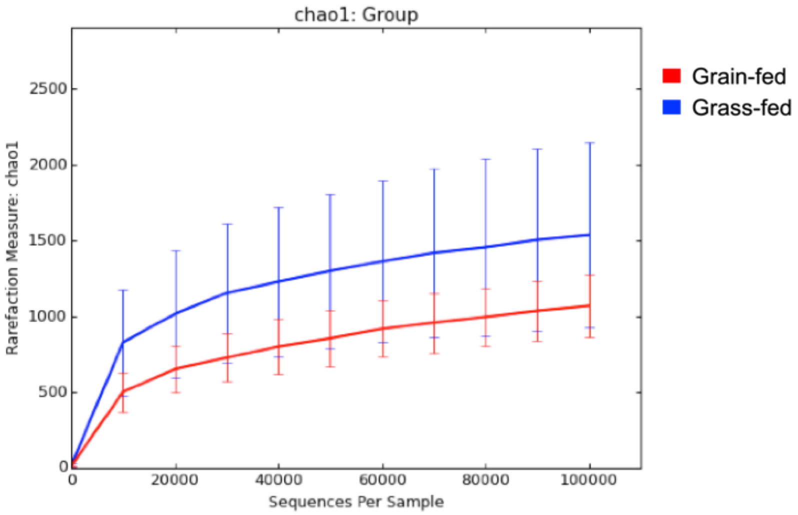

Figure S4 Rarefaction curves of the jejunal bacterial communities based on Chao1 values of grass-fed (blue) and grain-fed (red) groups.

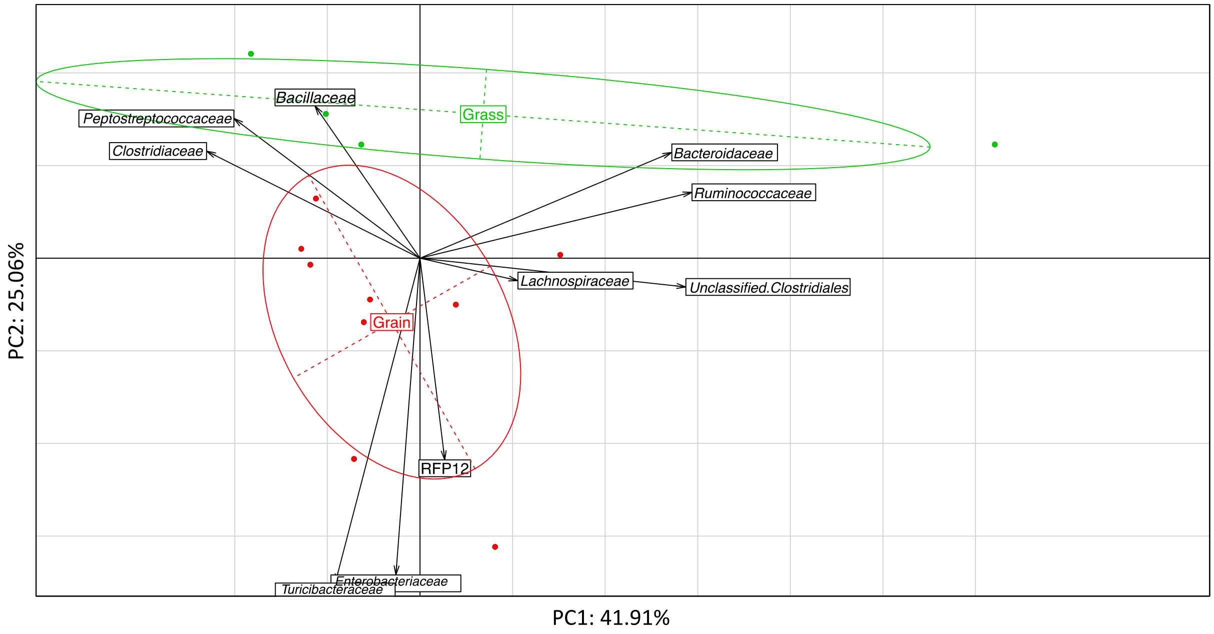

Figure S5 Ordination biplots of PCA on the jejunal bacteria. Arrows display the directions and relative importance of the top 10 abundant families. The variability in the jejunal bacterial communities is explained on x-axis with 39.91% and on y-axis with 20.05%. “d” on the top right of the figure stands for “dimension”.

Table S3 Bile acids in the jejunum, including those without significant differences in concentrations between two dietary groups ($p > 0.05$). The concentration unit showed as μmol/ml.

**Bile acids**	**Grain-fed**	**Grass-fed**	***p*-value**
Apocholic acid	0.043 ± 0.028	0.038 ± 0.043	0.2225
Dehydrolithocholic acid	0.032 ± 0.008	0.036 ± 0.007	0.4295
Deoxycholic acid	0.828 ± 0.056	0.774 ± 0.076	0.2713
Glycochenodeoxycholic acidt	19.651 ± 12.122	16.631 ± 3.081	0.2263
Glycohyodeoxycholic acid	0.021 ± 0.009	0.018 ± 0.005	0.5619
Ш-muricholic acid	0.07 ± 0.009	0.073 ± 0.018	0.7718
Taurochenodexycholic acid	15.947 ± 7.29	14.868 ± 2.207	0.9601
Tauro-α-muricholic acid	0.018 ± 0.009	0.017 ± 0.008	0.8729
Tauro-Ш-muricholic acid	0.164 ± 0.145	0.143 ± 0.061	0.7188
Ursodeoxycholic acid	0.211 ± 0.028	0.201 ± 0.012	0.7718
α-Muricholic acid	0.062 ± 0.029	0.078 ± 0.033	0.6384

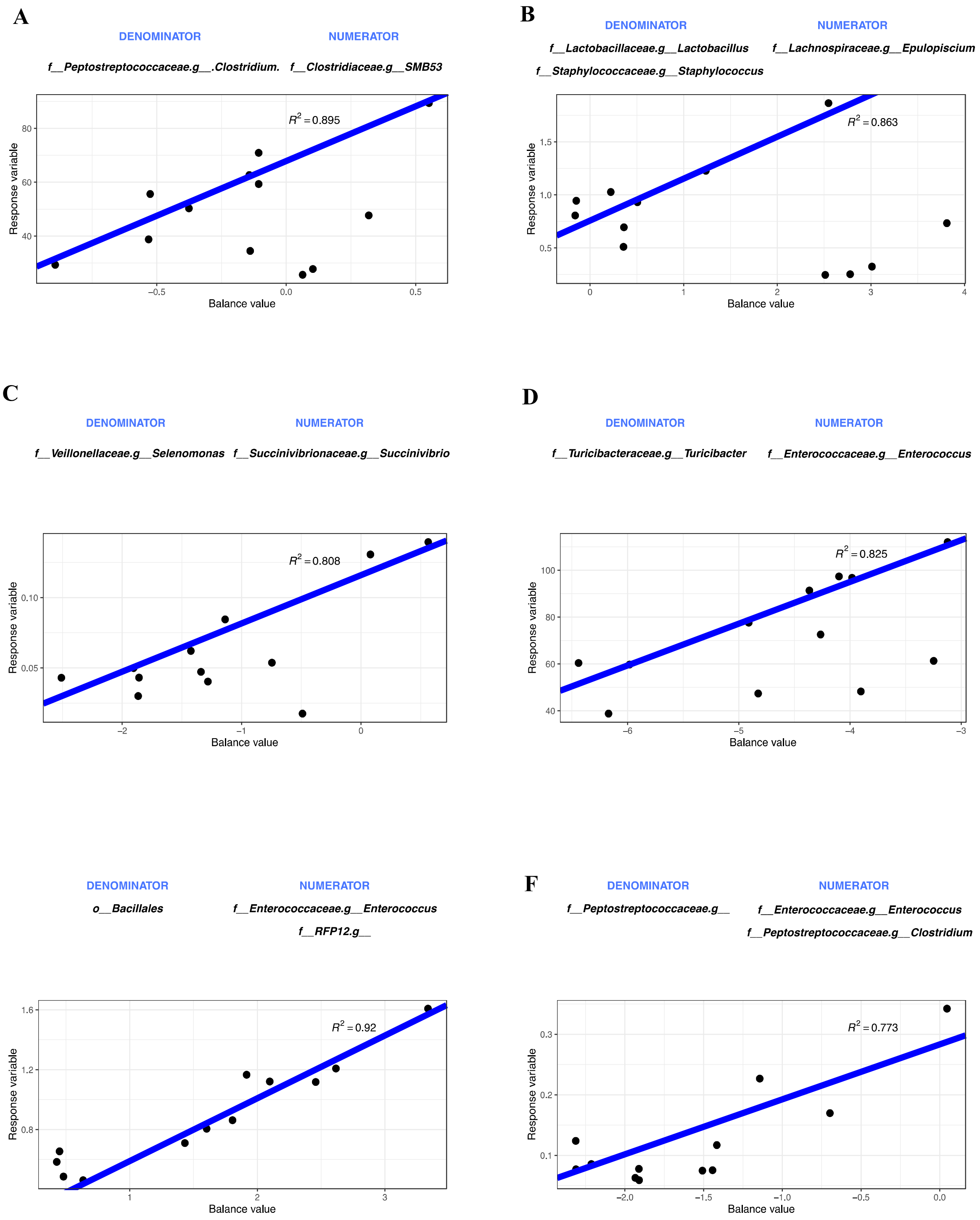

Figure S6 The global balances predictive for the levels of secondary bile acids. The regression model was analyzed for each type of secondary bile acids. Microbiome signatures, including taxa in both denominator and numerator, were specified at the top of each figure. (A) Glycodeoxycholic_acid. (B) Glycolithocholic_acid. (C) Glycoursodeoxycholic_acid. (D) Taurodeoxycholic_acid. (E) Taurolithocholic_acid. (F) Total of tauroursodeoxycholic/taurohyodeoxycholic acid.
